# High Glucose Treatment Induces Nuclei Aggregation of Microvascular Endothelial Cells via the *foxo1a*-*klf2a* Pathway

**DOI:** 10.1161/ATVBAHA.124.321719

**Published:** 2025-01-30

**Authors:** Xiaoning Wang, Xinyi Kang, Bowen Li, Changsheng Chen, Liping Chen, Dong Liu

**Affiliations:** 1 Research Center of Clinical Medicine, Affiliated Hospital (X.W., D.L.), Nantong University, China.; 2 School of Life Science, Nantong Laboratory of Development and Diseases, The Key Laboratory of Neuroregeneration of Jiangsu and Ministry of Education, Co-Innovation Center of Neuroregeneration (B.L., C.C., D.L.), Nantong University, China.; 3 Obstetrics and Gynecology Department, The Second Affiliated Hospital of Nantong University, China (X.K., L.C., D.L.).

**Keywords:** animals, biology, endothelial cells, glucose, humans

## Abstract

**BACKGROUND:**

Hyperglycemia is a major contributor to endothelial dysfunction and blood vessel damage, leading to severe diabetic microvascular complications. Despite the growing body of research on the underlying mechanisms of endothelial cell (EC) dysfunction, the available drugs based on current knowledge fall short of effectively alleviating these complications. Therefore, our endeavor to explore novel insights into the cellular and molecular mechanisms of endothelial dysfunction is crucial for the field.

**METHODS:**

In this study, we performed a high-resolution imaging and time-lapse imaging analysis of the behavior of ECs in *Tg(kdrl:ras-mCherry::fli1a:nGFP*) zebrafish embryos upon high glucose treatment. Genetic manipulation and chemical biology approaches were utilized to analyze the underlying mechanism of high glucose–induced nuclei aggregation and aberrant migration of zebrafish ECs and cultured human ECs. Bioinformatical analysis of single-cell RNA-sequencing data and molecular biological techniques was performed to identify the target genes of *foxo1a*.

**RESULTS:**

In this study, we observed that the high glucose treatment resulted in nuclei aggregation of ECs in zebrafish intersegmental vessels. Additionally, the aberrant migration of microvascular ECs in high glucose–treated embryos, which might be a cause of nuclei aggregation, was discovered. High glucose induced aggregation of vascular endothelial nuclei via *foxo1a* downregulation in zebrafish embryos. Then, we revealed that high glucose resulted in the downregulation of *foxo1a* expression and increased the expression of its direct downstream effector, *klf2a*, through which the aberrant migration and aggregation of vascular endothelial nuclei were caused.

**CONCLUSIONS:**

High glucose treatment caused the nuclei of ECs to aggregate in vivo, which resembles the crowded nuclei of ECs in microaneurysms. High glucose suppresses *foxo1a* expression and increases the expression of its downstream effector, *klf2a*, thereby causing the aberrant migration and aggregation of vascular endothelial nuclei. Our findings provide a novel insight into the mechanism of microvascular complications in hyperglycemia.

**Highlights**

- We first discovered that the high glucose treatment resulted in nuclei aggregation of endothelial cells in zebrafish intersegmental vessels, which resembles the crowded nuclei of endothelial cells in microaneurysms.
- Additionally, we observed the aberrant migration of microvascular endothelial cells in high glucose–treated embryos, which might be a cause of nuclei aggregation.
- Furthermore, through the analysis of single-cell sequencing data and molecular experiments, we revealed that high glucose suppresses foxo1a expression and increases the expression of its downstream effector, *klf2a*, thereby causing the aberrant migration and aggregation of vascular endothelial nuclei.
- Our findings provide a novel insight into the mechanism of microvascular complications in hyperglycemia.

Diabetic microvascular complications include diabetic retinopathy, diabetic nephropathy, and diabetic neuropathy,^1^ which might be potentially caused by tissue exposure to chronic hyperglycemia. High blood glucose levels harm endothelial cells (ECs), leading to endothelial dysfunction, resulting in microvascular hyperplasia, vascular lumen narrowing, and other pathological manifestations. The molecular mechanism of endothelial dysfunction in diabetes is complex, involving multiple factors, such as oxidative stress,^2–5^ activation of PKC (protein kinase C),^6,7^ overexpression of growth factors,^8–10^ nonenzymatic glycation of proteins,^11,12^ impaired insulin activation of PIP-3 kinase,^13–15^ and others. While cumulating studies have shed light on the underlying mechanisms of EC dysfunction, there remains a significant knowledge gap regarding the causes and mechanisms of diabetic microvascular complications. Notably, the currently available drugs cannot relieve these vascular diseases satisfactorily.^16–21^ Therefore, further exploration of the cellular and molecular drivers of endothelial dysfunction is crucial for developing effective therapeutic strategies for diabetic microvascular disease.

Zebrafish is an advantageous model and has been widely used in vascular research. Zebrafish embryos are optically transparent, allowing high-resolution live imaging of blood vessel development and pathological processes.^22^ Due to its similar glucose metabolism pathways to humans, the zebrafish is also an emerging disease model organism for research on diabetes and its vascular complications.^23^ Moreover, the genetic manipulation strategies of zebrafish are relatively simple, making it convenient for gene loss of function and gain of function.^24–27^ The in vivo imaging analysis of the zebrafish model offers an opportunity to discover novel behaviors of microvascular ECs under hyperglycemia conditions.

In the present study, we show that high glucose treatment induces the aggregation of vascular endothelial nuclei in zebrafish embryos’ intersegmental vessels (ISVs). Furthermore, we revealed that high glucose induced EC nuclei aggregation by downregulating *foxo1a* and increasing the expression of its direct downstream effector, *klf2a*. This study showed for the first time that high glucose causes the aggregation of endothelial nuclei, providing novel insights into the mechanism of microvascular complications in hyperglycemia.

## Materials and Methods

The data that support the findings of this study are available from the corresponding author upon reasonable request. Moreover, the single-cell RNA-sequencing data produced in the course of this study have been deposited into the Gene Expression Omnibus database under the accession number GSE276251.

### Ethics Approval

All zebrafish experimentation was performed following the National Institutes of Health guidelines for the care and use of laboratory animals (http://oacu.od.nih.gov/regs/index.htm) and ethically approved by the Administration Committee of Experimental Animals, Jiangsu Province, China (approval ID: 20180905-002).

### Zebrafish

*Tg(kdrl:ras-mCherry*) and *Tg(fli1a:nGFP*) transgenic zebrafish were used in this study, in which EC membrane was labeled with mCherry and endothelial nucleus was labeled with GFP. Zebrafish embryos for whole-mount in situ hybridization were obtained through natural mating (AB line). Zebrafish embryos were treated with 0.2 mmol/L 1-phenyl 2-thiourea (Sigma-Aldrich; P7629) after 24 hours post-fertilization (hpf) to prevent pigment formation. According to our previous work, all of these embryos and adult fish were maintained under standard conditions.^28^

### Drug Treatment

Zebrafish embryos with developmental defects or delays were removed at 8 hpf and then raised in 12-well plates at 10 embryos per well at 28.5 °C. Embryos were treated with 300 mmol/L D-glucose (Sigma; G7021) at different time windows. Isotonic maltose was used as a negative control, and the osmolarity was calculated using the formula reported previously.^29^ AS1842856 (MCE; HY-100596) powder was dissolved in dimethyl sulfoxide into a 10-mmol/L stock solution, stored at −80 °C, and used at 1 µmol/L for zebrafish embryos and 0.1 µmol/L for human umbilical vein ECs (HUVECs). The same concentration of dimethyl sulfoxide was used as a negative control.

### Glucose Concentration Measurement

Glucose concentration in the embryos was measured as described in our previous work.^30^ Briefly, embryos were selected and transferred to 24-well plates (10 embryos per well) and immersed in high glucose solution at 48 hpf. For glucose concentration measurement, embryos (n=20) were transferred to a new 1.5-mL tube, rinsed 3× with 1×PBS, and then discarded the PBS as much as possible. Subsequently, embryos were homogenized using a hand homogenizer and centrifuged at 14 000*g* for 10 minutes, and 1.5 μL of the supernatant was used to measure the total free-glucose level using a glucometer (Baye; 7600P).

### Whole-Mount In Situ Hybridization

The whole-mount in situ hybridization and the preparation of an antisense RNA probe were performed as described in the previous protocol.^31^ The *klf2a* cDNA fragment was cloned with the specific primers (Table S1) using the wild-type embryo (AB) cDNA library. The antisense probe was synthesized using the in vitro DIG-RNA Labeling Transcription Kit (Roche; 11175025910) with linearized pGEM-T easy vector containing *klf2a* fragment as the templates. The synthesized probe was purified with LiCl (Invitrogen; AM9480) and diluted to 1 ng/µL for hybridization. Zebrafish embryos at 72 hpf were collected and fixed with 4% paraformaldehyde overnight at 4 °C and then dehydrated with gradients of methanol and stored at −20 °C in 100% methanol for subsequent analysis. The hybridization result was detected with anti-DIG-AP antibody (1:2000; Roche; 11093274910) and NBT/BCIP (1:500; Roche; 11681451001). After hybridization, images of the embryos were captured with an Olympus MVX10 stereomicroscope.

### RNA Isolation, Reverse Transcription, and Quantitative RT-PCR

Total RNA was isolated with TRIzol reagent (Invitrogen; 15596026) and stored at −80 °C. Afterward, the cDNA was synthesized using the HiScript III First Strand cDNA Synthesis Kit (Vazyme; R312-01) and stored at −20 °C. Quantitative PCR was performed using the Taq Pro Universal SYBR qPCR Master Mix (Vazyme; Q712-02) on the basis of the manufacturer’s instructions. For the quantification of mRNA levels, the ΔΔCT method was used. Primers for real-time PCR analysis are listed in Table S1.

### Single-Cell RNA Sequencing and Gene Expression Profile Analysis

Single-cell preparation and gene expression profile analysis were performed as described in our previous work.^30^ Briefly, the *Tg(fli1aEP:EGFP-CAAX*)^*ntu666*^ embryos were treated with 300 mmol/L glucose for 3 days and then collected and dissociated using 0.25% trypsin at 28.5 °C. EGFP-positive cells were enriched by fluorescence-activated cell sorting and then processed for RNA sequencing using the Chromium platform (10× Genomics). Cell Ranger 3.0.2 (https://github.com/10XGenomics/cellranger) was used to convert the raw sequencing data to a single-cell level gene count matrix. The clustering of single cells and the marker genes in each cluster were analyzed by Seurat 3.0 (https://satijalab.org/seurat/install.html).^32^ Additionally, the sctransform method^33^ was applied to remove technical variation, and ClusterProfiler^34^ was used to do the gene ontology enrichment analysis based on the marker genes of each cell cluster. Detailed information about the data processing can be found in this project’s source code (https://github.com/gangcai/ZebEndoimmune).

### Plasmid Construction and Microinjection

The coding sequence of *klf2a* and the sequence of *foxo1a*-6×His were synthesized and inserted, respectively, into *hsp70l:MCS-P2A-mCherry* vector by Genewiz Co, Ltd (Suzhou, China). The sequence of *klf2a* (dominant negative) was designed referring to the previous work^35^ and added to the C terminus of mApple fluorescent protein through gene synthesis to get the construction of *Tg(hsp70l:mApple-klf2a-DN*). For microinjection, 60 ng Tol2 transposase and 75 ng plasmid were mixed well in 5 µL water, respectively, and a 2-nL mixture was microinjected into zebrafish embryos at the 1-cell stage. For heat shock treatment, embryos were transferred to a 1.5-mL centrifuge tube, incubated for 1 hour at 37 °C, and then raised in the dish with new egg water at 28.5 °C.

### Chromatin Immunoprecipitation–PCR

Embryos injected with *Tg(hsp70l:foxo1a-6×His-P2A-mCherry*) were collected at 72 hpf after heat shock treatment. The chromatin immunoprecipitation (ChIP)–PCR assay was performed using the ChIP Assay Kit (Millipore; 3753379) according to the manufacturer’s instructions. The genomic DNA crossed with Foxo1a (forkhead box protein O1a) protein was immunoprecipitated by Ni NTA Beads (Smart-Lifesciences; SA004100) following the manufacturer’s instructions. Antibo dy against IgG was used as a negative control. The semiquantitative PCR was performed with KODfx (KFX-101; Toyobo) at the following conditions: 94 °C for 5 minutes; 35 cycles of 98 °C for 10 s, 55 °C for 30 s, 68 °C for 10 s, and 68 °C for 10 minutes. The PCR primers used for the predicted binding sites are listed in Table S1.

### Luciferase Reporter Assay

Luciferase reporter assay was performed according to our previous work.^30^ Briefly, zebrafish *klf2a* promoter fragment with predictive Foxo1a-binding site was cloned and inserted into pGL4.10 basic vector by KpnI and EcoRV. Next, 50 pg of PGL4.10 vectors, 1 pg of PGL4.74 vectors, and 50 pg of *foxo1a* mRNA or 20 pg of *foxo1a* morpholino were coinjected into zebrafish embryos at the 1-cell stage, and then, embryos were harvested at 24 hpf to measure their luciferase activity according to the manufacturer’s protocols (Promega).

### Cell Culture and Wound-Healing Assay

HUVECs and human retinal microvascular ECs were cultured according to the protocols described previously.^36^ For the wound-healing assay, a dish with a defined cell-free gap (Ibidi; 80206) was used to create the gap following the manufacturer’s instructions. Cells were treated with 1 μg/mL mitomycin C (Sigma-Aldrich; M5353) for 1 hour before the experiment to inhibit the effects of cell proliferation. Images were captured at 0, 6, 12, and 18 hours after drug treatment with the Olympus IX73 microscope, and the scratch area was measured with the ImageJ software.

### Confocal Imaging and Quantitative Analysis

For confocal imaging, zebrafish embryos were anesthetized with egg water/0.16 mg/mL tricaine (Sigma-Aldrich; A5040) and embedded in 0.7% low–melting point agarose. Live imaging was performed with the Nikon A1R confocal microscope. For time-lapse imaging, embryos were placed on a platform at a constant temperature of 28.5 °C. All of the data were measured with the ImageJ software. For time-lapse data, using the dorsal aorta as a reference, the distance of the endothelial nucleus migration was measured by the shift of the nucleus in the before and after images. Nuclei nearest neighbor distance was quantified using the Fiji ND plugin according to the protocol described previously.^37,38^

### Statistical Analysis

For statistical analysis, data normality was determined first using the Shapiro-Wilk test. Unless otherwise stated, data with normal distribution were analyzed using unpaired Student *t* tests (data with 2 groups), 1-way ANOVA (data with >2 groups), and 2-way ANOVA (data with multiple variables). All data are presented as mean±SEM, and *P*<0.05 was considered to be statistically significant.

## Results

### High Glucose Treatment Induced the Aggregation of Vascular Endothelial Nuclei in the ISVs of Zebrafish Embryos

**Figure 1.**
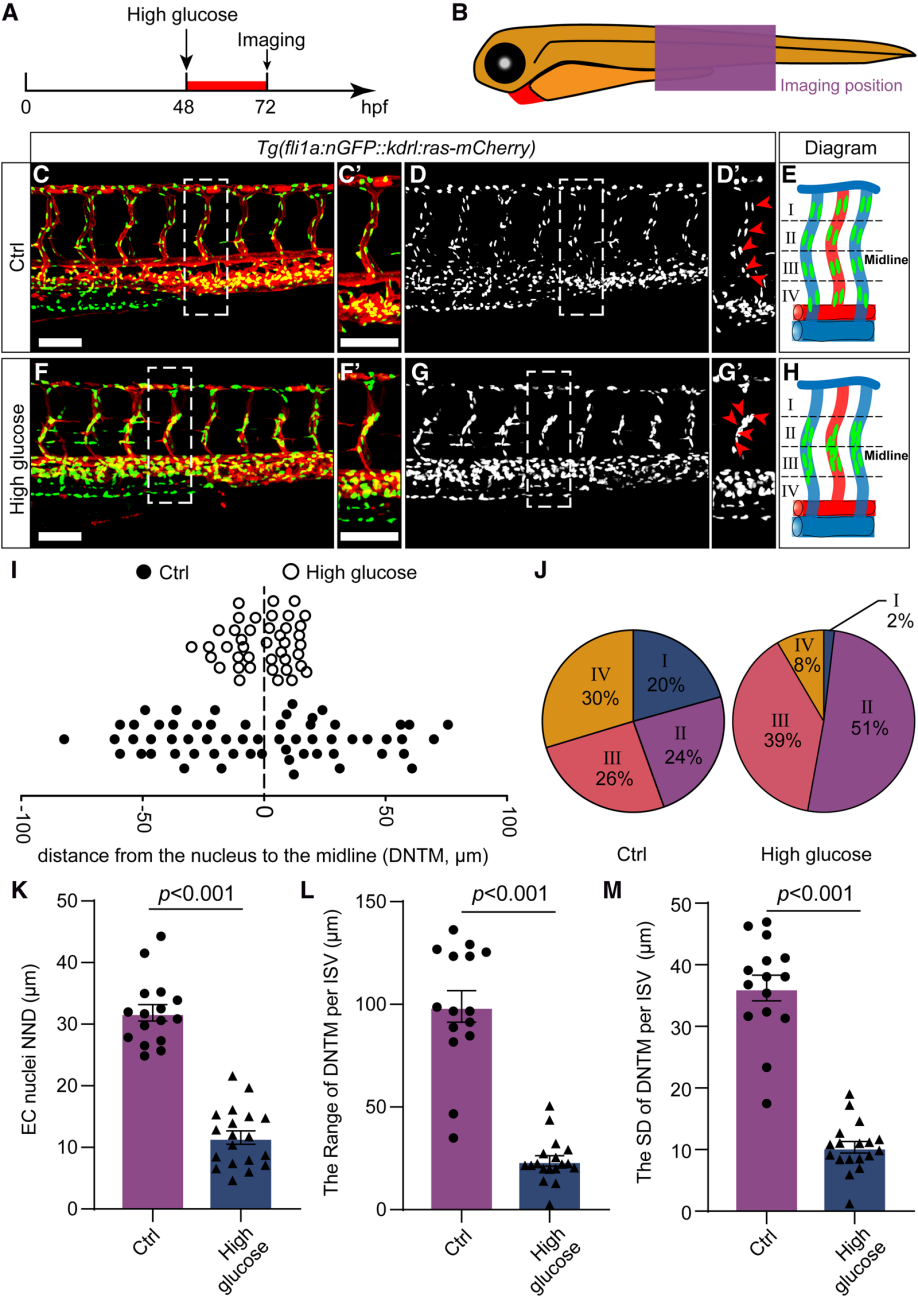
**High glucose treatment induced the aggregation of vascular endothelial cell (EC) nuclei in the intersegmental vessels (ISVs) of zebrafish embryos. A** and **B**, Schematic diagram showing the high glucose treatment timeline and confocal imaging region. **C** through **G**, Confocal imaging analysis of control (Ctrl) and high glucose–treated *Tg(fli1a:nGFP::kdrl:ras-mCherry*) embryos at 72 hours post-fertilization (hpf). **C′** through **G′**, The magnifications of the red dotted boxes in **C** through **G**, respectively. The arrowheads indicate the EC nuclei in the ISV. **I**, The distance from the EC nuclei to the midline (DNTM) in ISVs in Ctrl and high glucose–treated embryos. **J**, The proportion of EC nuclei in ISVs in the 4 zones is shown in **E** and **H**. **K**, Statistics of the nearest neighbor distance (NND) of EC nuclei in Ctrl embryos (n=16) and high glucose–treated embryos (n=19). *t* test. **L** and **M**, Statistics of the range and SD of DNTM in Ctrl embryos (n=15) and high glucose–treated embryos (n=18). *t* test. Scale bars, 100 µm.

**Figure 2.**
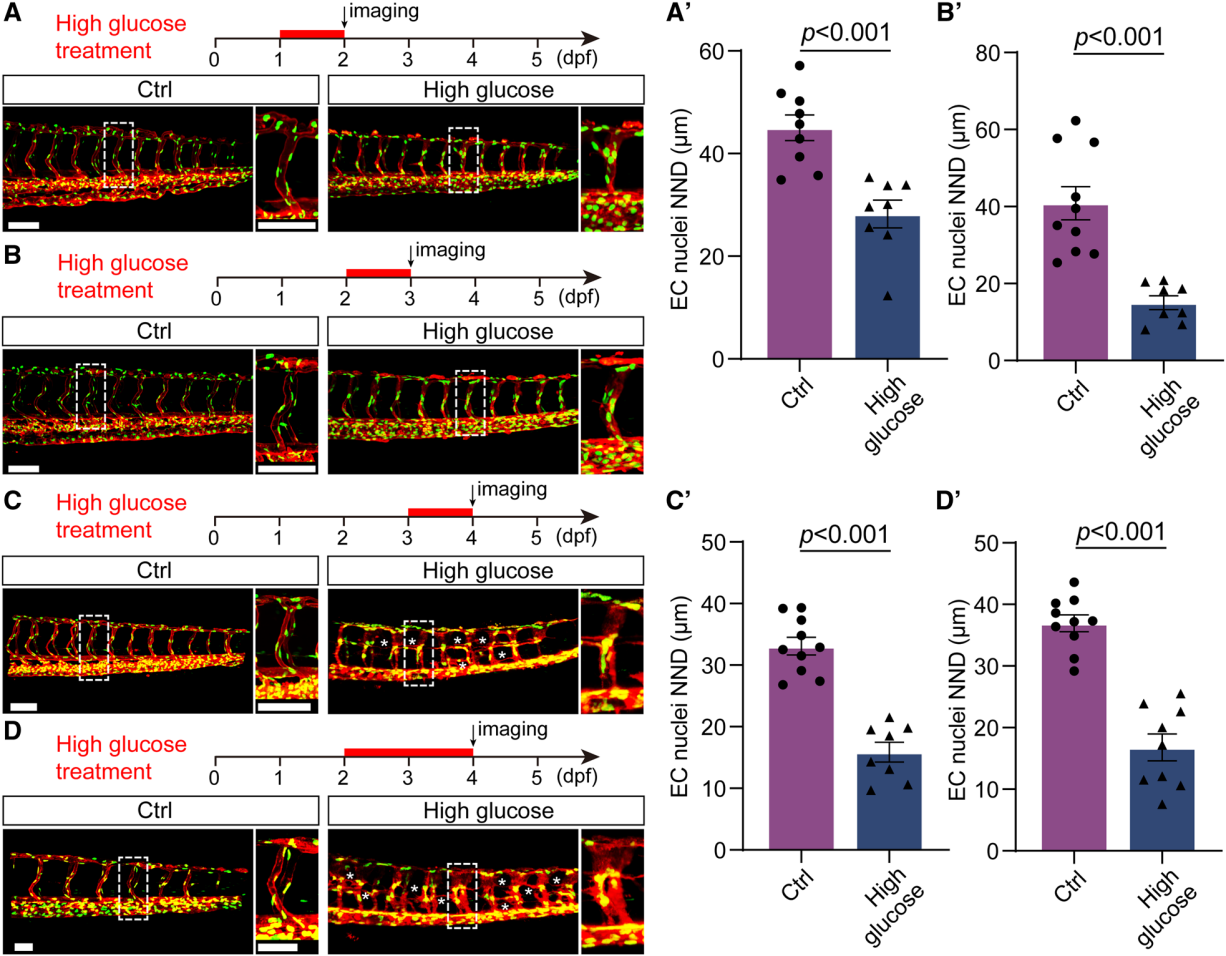
**The aggregation of endothelial cell (EC) nuclei in zebrafish embryos was induced by high glucose treatment at different time windows. A** through **D**, Schematic diagrams of different time windows of high glucose treatment and confocal images. The **right** panels are the magnifications of the white dotted boxes. **A′** through **D′**, Statistics of the EC nuclei nearest neighbor distance (NND) in different time windows in **A** through **D**, respectively. n=9 control (Ctrl; **A′**) and n=8 high glucose (**A′**), n=10 Ctrl (**B′**) and n=8 high glucose (**B′**), n=10 Ctrl (**C′**) and n=8 high glucose (**C′**), n=10 Ctrl (**D′**) and n=9 high glucose (**D′**). *t* test. Scale bars, 100 µm.

In the previous work, we have reported that high glucose treatment could cause ISV hyperplasia in zebrafish embryos.^30^ To further examine the effects of high glucose on ECs, we treated zebrafish embryos with a high dose of D-glucose (300 mmol/L) for a short period. We subsequently measured the glucose concentration in the embryos, and the results showed that the glucose concentration in the embryos treated with high glucose was significantly higher than that in the control group (Figure S1). Confocal imaging analysis of *Tg(kdrl:ras-mCherry::fli1a:nGFP*) embryos revealed that the treatment of high glucose from 48 to 72 hpf caused endothelial nucleus aggregation in almost all ISVs of the embryos (Figure 1A through 1H). A similar phenotype was observed in zebrafish ocular vessel at 72 hpf (Figure S2). The nearest neighbor distance of EC nuclei and distance from the ECs nuclei to the midline were also reduced compared with control (Figure 1I and 1K). Subsequently, the ISVs were partitioned into 4 longitudinal zones, wherein the EC nuclei in the control group exhibited uniform distribution (Figure 1E and 1J). In contrast, those in the treated group were predominantly concentrated in zones II and III (Figure 1H and 1J). The range and SD of the distance from the EC nuclei to the midline in the treated embryos were also smaller than those in the control siblings (Figure 1L and 1M). To rule out the effect of osmotic pressure, we treated zebrafish embryos with isotonic maltose. The results showed that high maltose treatment did not cause endothelial nucleus aggregation (Figure S3). Furthermore, we conducted the treatments on the embryos during various time intervals and observed that exposure to high glucose for either 1 day (≈24–48, ≈48–72, and ≈72–96 hpf) or 2 days (≈48–96 hpf), all led to the aggregation of endothelial nuclei and decreased nearest neighbor distance in the ISVs of zebrafish embryos (Figure 2A through 2D) in comparison to control (Figure 2A′ through 2D′). These results suggest that the endothelial nuclei in ISVs become more concentrated after high glucose treatment.

### High Glucose Treatment Causes Excessive Migration of Vascular Endothelial Nuclei in Zebrafish Embryos

**Figure 3.**
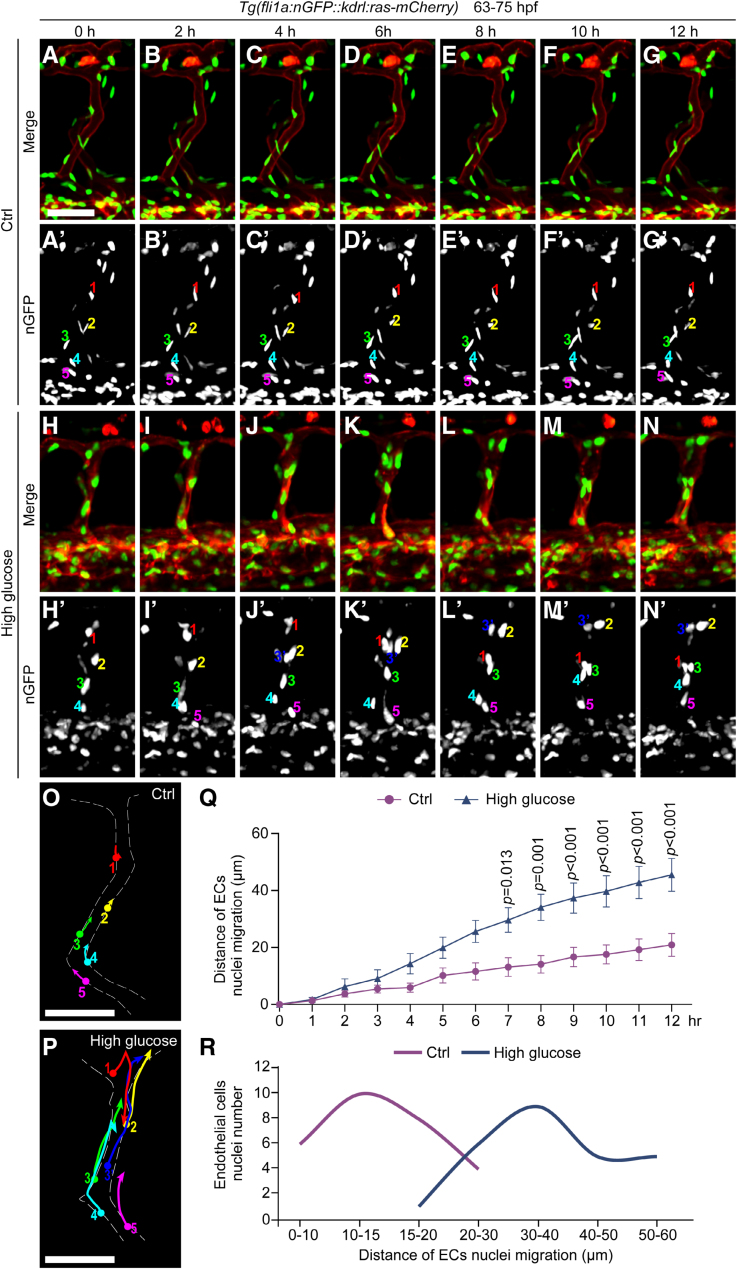
**High glucose treatment caused excessive migration of vascular endothelial cell (EC) nuclei in intersegmental vessels (ISVs) of zebrafish embryos. A** through **N**, Still images from in vivo time-lapse imaging analysis of *Tg(fli1a:nGFP::kdrl:ras-mCherry*) embryos from 63 to 75 hours post-fertilization (hpf). **A′** through **N′**, The nGFP channel of the above panels, respectively. The numbers represent different EC nuclei in unilateral ISVs. **O** and **P**, Diagrams of the migration routes of EC nuclei labeled in **A′** through **N′**. **Q**, Statistics of the distance of EC nuclei migration at different time stages in control (Ctrl) embryos (n=8) and high glucose–treated embryos (n=8). Two-way ANOVA. **R**, The number of EC nuclei in different migration distances of Ctrl embryos and high glucose–treated embryos. Scale bars, 50 µm.

To further investigate the cellular effects of high glucose treatment on the ECs of zebrafish embryos, we performed time-lapse imaging to surveil the behavior of EC nuclei from 63 to 75 hpf (Figure 3A through 3N; Videos S1 and S2). We first checked the proliferation of ECs; the results showed no significance between control and high glucose–treated group (Figure S4). Compared with the control group, the EC nuclei in embryos subjected to high glucose treatment exhibited hyperactivity. In contrast, the EC nuclei in the control embryos remained relatively stationary (Figure 3A′ through 3G′ and 3O), while those in the treated embryos displayed excessive migration (Figure 3H′ through 3N′ and 3P). The distance of EC nuclei migration in the treated embryos was significantly greater than that observed in the control embryos (Figure 3Q). Furthermore, the analysis of the number of EC nuclei within different migration distance ranges revealed that the migration distance of EC nuclei in control embryos was predominantly within the range of ≈10 to 15 µm. In contrast, in the treated embryos, it was primarily within the range of ≈30 to 40 µm, ≈3× greater (Figure 3R). The results suggested that high glucose treatment led to excessive migration of EC nuclei in zebrafish embryos.

### High Glucose–Induced Aggregation of Vascular Endothelial Nuclei via *foxo1a* Downregulation in Zebrafish Embryos

**Figure 4.**
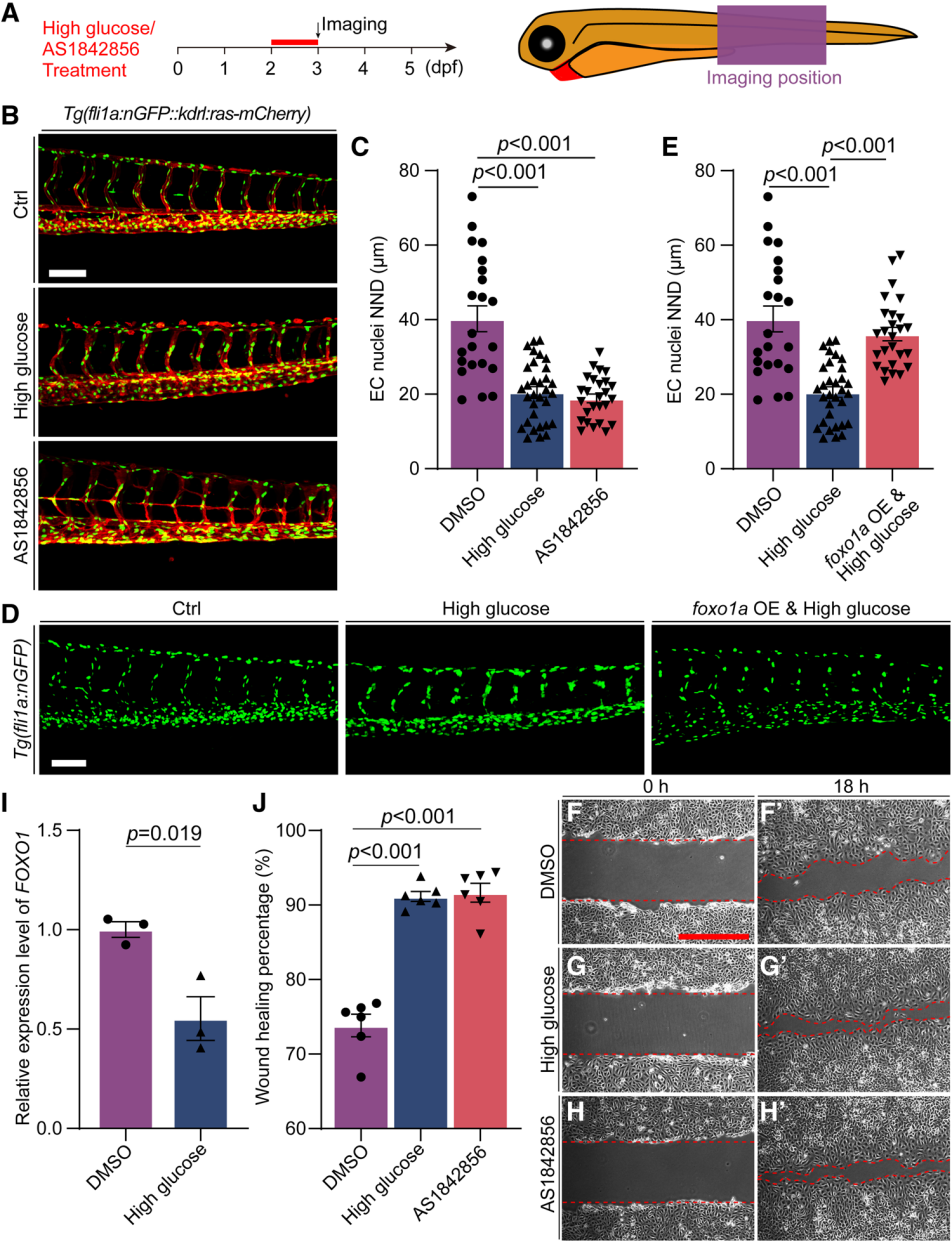
**Foxo1 (forkhead box protein O1) deficiency promoted human retinal microvascular endothelial cell (HRMEC) migration and resulted in the aggregation of endothelial cell (EC) nuclei in zebrafish embryos. A**, Schematic diagram of drug treatment time window and confocal imaging region. **B**, Confocal imaging analysis of control (Ctrl), high glucose–treated, and AS1842856-treated *Tg(fli1a:nGFP::kdrl:ras-mCherry*) embryos at 72 hours post-fertilization (hpf). **C**, Statistics of the EC nuclei nearest neighbor distance (NND) in Ctrl (n=22), high glucose–treated (n=32), and AS1842856-treated embryos (n=26). *t* test. **D**, Confocal imaging analysis of EC nuclei in ISVs in the Ctrl embryos, high glucose–treated embryos, and *Tg(hsp70l:foxo1a-6×His-P2A-mCherry*) and high glucose–treated embryos at 72 hpf. **E**, Statistical analysis of the EC nuclei NND in the Ctrl embryos (n=22), high glucose–treated embryos (n=32), and *Tg(hsp70l:foxo1a-6×His-P2A-mCherry*) and high glucose–treated embryos (n=26) at 72 hpf. One-way ANOVA. **F** through **H′**, The results of the wound-healing assay showed that high glucose and AS1842856 treatment promotes HRMEC migration, in comparison with the dimethyl sulfoxide (DMSO)–treated group. **I**, Real-time PCR analysis of *FOXO1* expression in HRMECs treated with DMSO and high glucose. Each dot represents data from an independent experiment (n=3). *t* test. **J**, Statistical analysis of the wound-healing percentage in the DMSO, high glucose–treated, and AS1842856-treated groups. Each dot represents data from an independent experiment (n=6). *t* test. Scale bars, 100 µm. nGFP indicates green fluorescent protein for nuclei.

Our previous work found that high glucose–induced excessive sprouting angiogenesis in zebrafish was mediated by the downregulation of *foxo1a*. It has been reported that FOXO1 was involved in the process of EC migration.^39^ Hence, the hypothesis that high glucose treatment induced the aggregation of vascular endothelial nuclei was also mediated by *foxo1a*. To verify our speculation, control zebrafish embryos were treated with AS1842856, a cell-permeable inhibitor that blocks the transcription activity of FOXO1.^40^ The results revealed that the inhibition of Foxo1 also resulted in the aggregation of EC nuclei in ISVs, resembling the phenotype observed in high glucose–treated embryos. Moreover, the nearest neighbor distance of EC nuclei in AS1842856-treated embryos was significantly lower compared with that of control embryos (Figure 4A through 4C). To further confirm the effect of *foxo1a*, we performed a rescue experiment by overexpressing *foxo1a* in zebrafish embryos. The *Tg(hsp70l:foxo1a-6×His-P2A-mCherry*) constructs and Tol2 transposase mRNA were comicroinjected into 1-cell-stage embryos and followed by heat shock treatment at 24 hpf to overexpress *foxo1a*. Next, the control and embryos overexpressing *foxo1a* were treated with high glucose from 48 to 72 hpf. The results showed that overexpression of *foxo1a* reduced the aggregation of vascular endothelial nuclei induced by high glucose (Figure 4D and 4E).

We also performed Foxo1 inhibition and wound-healing assay in HUVECs and human retinal microvascular ECs. The cells were treated with 0.1 μmol/L AS1842856 for 24 hours, and the wound-healing assay result showed that Foxo1 inhibition promoted both human retinal microvascular EC and HUVEC migration compared with dimethyl sulfoxide treatment (Figure 4F through 4H; Figure S5). These results suggest that high glucose may induce the nuclei aggregation and migration of ECs in zebrafish embryos through *foxo1a*.

### Klf2a Was Significantly Upregulated in Arterial and Capillary ECs

**Figure 5.**
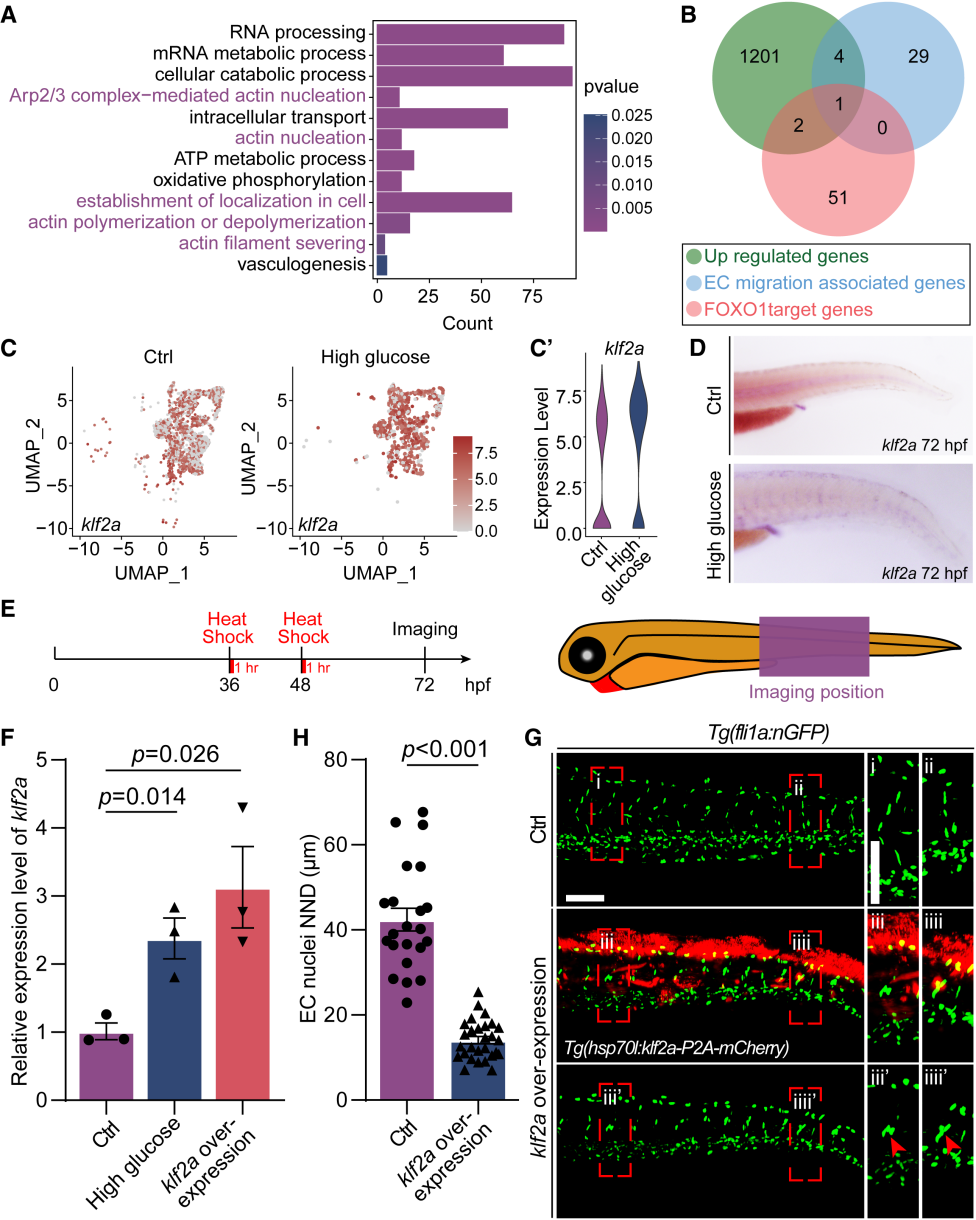
**Klf2a (Krüppel-like transcription factor 2a) was involved in the nuclei aggregation induced by high glucose treatment. A**, GO analysis of 1201 upregulated genes in arterial and capillary endothelial cells (ECs). **B**, Venn diagram showing the overlap between FOXO1 (forkhead box protein O1) target genes, EC migration–associated genes, and significantly upregulated genes. **C**, The feature plot of *klf2a* of control (Ctrl) and high glucose group in arterial and capillary ECs. **C′**, The violin plot of *klf2a* of Ctrl and high glucose group in arterial and capillary ECs. **D**, Whole-mount in situ hybridization analysis of *klf2a* in Ctrl and high glucose–treated embryos. **E**, Schematic diagram of heat shock treatment and confocal imaging region. **F**, Real-time PCR analysis of *klf2a* expression in Ctrl, high glucose–treated, and *klf2a* overexpressed embryos. Each dot represents data from an independent experiment (n=3). *t* test. **G**, Confocal imaging analysis of Ctrl embryos and *Tg(hsp70l:klf2a-P2A-mCherry*) embryos at 72 hours post-fertilization (hpf). The **right** panels (i, ii, iii, iiii, iii', iiii') are the magnifications of the red dotted boxes. Arrowheads indicate the EC nuclei. **H**, Statistics of the EC nuclei nearest neighbor distance (NND) in Ctrl (n=22) and *Tg(hsp70l:klf2a-P2A-mCherry*) embryos (n=28). *t* test. Scale bars, 100 µm. Arp2/3 indicates actin-related proteins-2/3; GO, gene ontology; PCR, polymerase chain reaction; and UMAP, uniform manifold approximation and projection.

To identify the potential downstream factors for nuclei aggregation and migration of ECs in the embryos treated with glucose, we analyzed and compared the differentially expressed genes in arterial and capillary ECs of control and glucose-treated ECs, which was described in our previous work.^30^ We performed GO analysis of significantly upregulated genes, and the results revealed that these genes were enriched in several biological processes, including cellular catabolic process, intracellular transport, establishment of localization in cells, and actin filament severing (Figure 5A). Subsequently, the upregulated genes were further intersected with FOXO1 target genes^41–43^ (Table S2) and EC migration–associated genes^39,44–48^ (Table S3), and we found *klf2a* (Figure 5B). Single-cell RNA-sequencing data revealed that *klf2a* was significantly upregulated in arterial and capillary ECs after high glucose treatment (Figure 5C and 5C′). The in situ hybridization experiment further confirmed the expression increase of *klf2a* following high glucose treatment (Figure 5D).

To verify whether the upregulation of *klf2a* led to the nuclei aggregation and migration of ECs in zebrafish embryos, we performed gain-of-function experiments targeting *klf2a*. we conducted an experiment with the overexpression of *klf2a*. We microinjected the *Tg(hsp70l:klf2a-P2A-mCherry*) constructs and Tol2 transposase mRNA into 1-cell-stage embryos, followed by heat shock treatment at 36 and 48 hpf (Figure 5E). The findings revealed that *klf2a* overexpression also results in nuclei aggregation and aberrant migration of ECs (Figure 5F and 5G; Video S3), which were consisted with the observed phenotype in high glucose–treated embryos.

### High Glucose–Induced Nuclei Aggregation and Migration Through the *foxo1a-klf2a* Pathway

**Figure 6.**
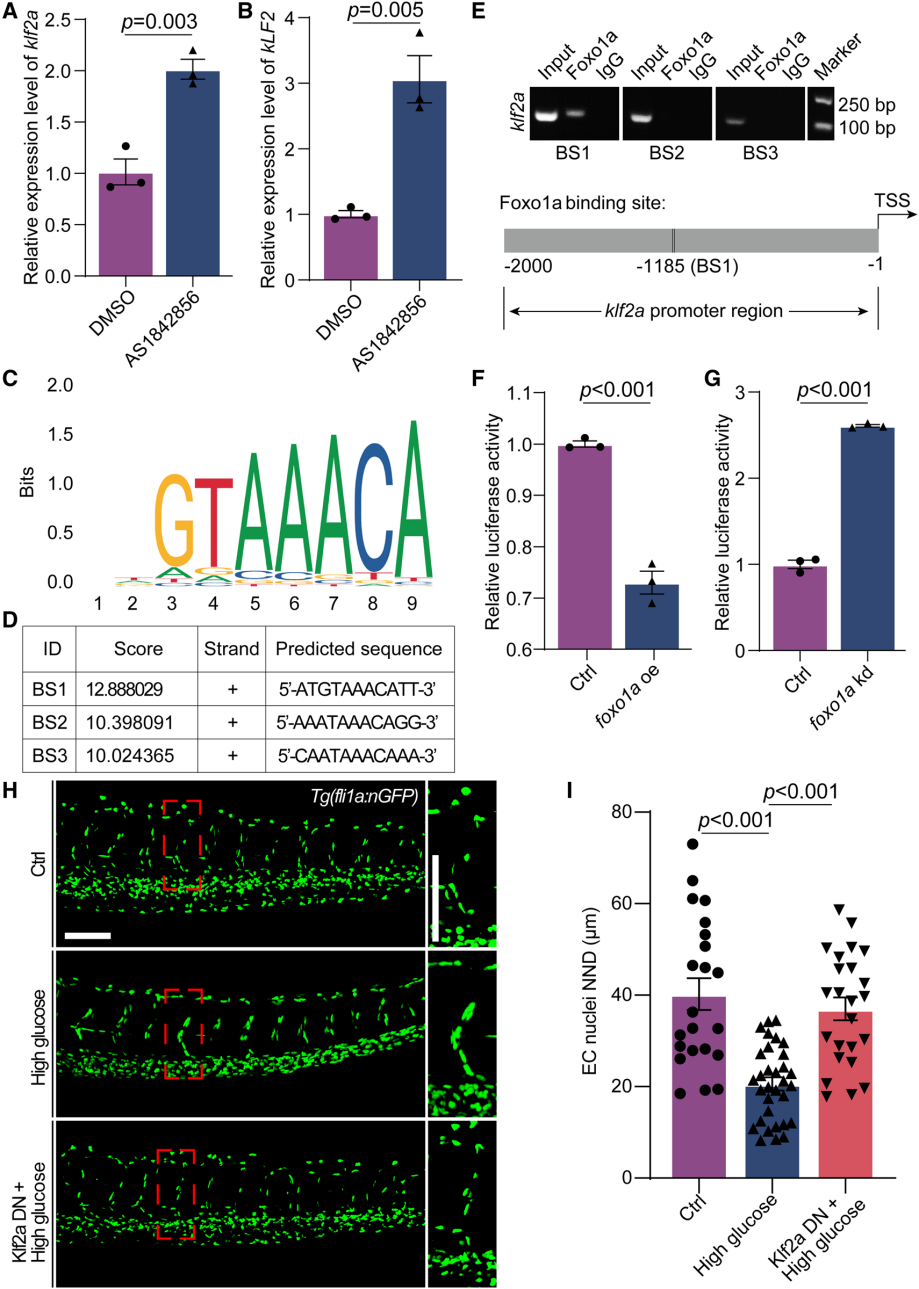
**High glucose treatment induced nuclei aggregation through *foxo1a-klf2a* signal in zebrafish embryos. A**, Real-time PCR analysis of *klf2a* expression in control (Ctrl) and AS1842856-treated embryos. Each dot represents data from an independent experiment (n=3). *t* test. **B**, Real-time PCR analysis of *KLF2* expression in human umbilical vein endothelial cells (ECs) treated with dimethyl sulfoxide (DMSO) and AS1842856. Each dot represents data from an independent experiment (n=3). *t* test. **C**, A potential Foxo1 (forkhead box protein O1)-binding sequence presented in JASPAR database. **D**, Three Foxo1a different candidate binding sites (BSs) upstream of the transcription start site of *klf2a* in zebrafish. **E**, Results of the chromatin immunoprecipitation–PCR assay demonstrated that the predicted sequence ATGTAAACATT is a Foxo1a BS of *klf2a* in zebrafish. Schematic diagram showing the Foxo1a BS in the *klf2a* promoter region. Input sonicated genomic DNA samples without immunoprecipitation as a positive control. IgG, sonicated, and IgG-immunoprecipitated genomic DNA samples as a negative control. **F** and **G**, Luciferase reporter activity in *foxo1a* overexpressed or knocked down embryos, respectively. Each dot represents data from an independent experiment (n=3). *t* test. **H**, Confocal imaging analysis of Ctrl embryos, high glucose–treated embryos, and *Tg(hsp70l:mApple-klf2a-DN*) and high glucose–treated embryos at 72 hours post-fertilization (hpf). **I**, Statistical analysis of the EC nuclei nearest neighbor distance (NND) in the Ctrl (n=22), high glucose–treated (n=32), and *Tg(hsp70l:mApple-klf2a-DN*) and high glucose–treated embryos (n=24) at 72 hpf. One-way ANOVA. Scale bars, 100 µm. DN indicates dominant negative; and PCR, polymerase chain reaction.

To examine whether *klf2a* is a downstream effector of Foxo1, we performed a series of experiments. We first investigated the impact of Foxo1 inhibition on *klf2a* expression in zebrafish embryos and HUVECs. As expected, qPCR analysis revealed that inhibition of Foxo1 by AS1842856 resulted in the upregulation of *KLF2* and *klf2a* expression in HUVECs and zebrafish embryos, respectively (Figure 6A and 6B). Next, we searched the FOXO1 target sequence through the JASPAR database (https://jaspar.genereg.net/) and identified 3 potential binding sites in the *klf2a* promoter region (Figure 6C and 6D). ChIP-PCR results showed that within 2 kb upstream of *klf2a* transcription start sites, a sequence of 5′-ATGTAAACATT-3′ at −1185 to −1195 nucleotides is a potential Foxo1a-binding site of zebrafish, suggesting that Foxo1a might bind to the promoters of *klf2a* and regulate its transcription (Figure 6E).

Additionally, we generated the *klf2a* dominant-negative construct *Tg(hsp70l:mApple-klf2a-DN*; Figure S6) and microinjected the construct and Tol2 mRNA into the embryos at 1-cell stage, followed by heat shock treatment at 36 and 48 hpf. Subsequently, the embryos were treated with high glucose, and their phenotypes were examined using confocal imaging analysis. The results demonstrated that Klf2a (Krüppel-like transcription factor 2a) deficiency significantly restored the EC nuclei distribution (Figure 6F and 6G). Collectively, these results suggest that high glucose treatment might induce the aggregation of ECs nuclei in zebrafish embryos via the *foxo1a-klf2a* pathway.

## Discussion

**Figure 7.**
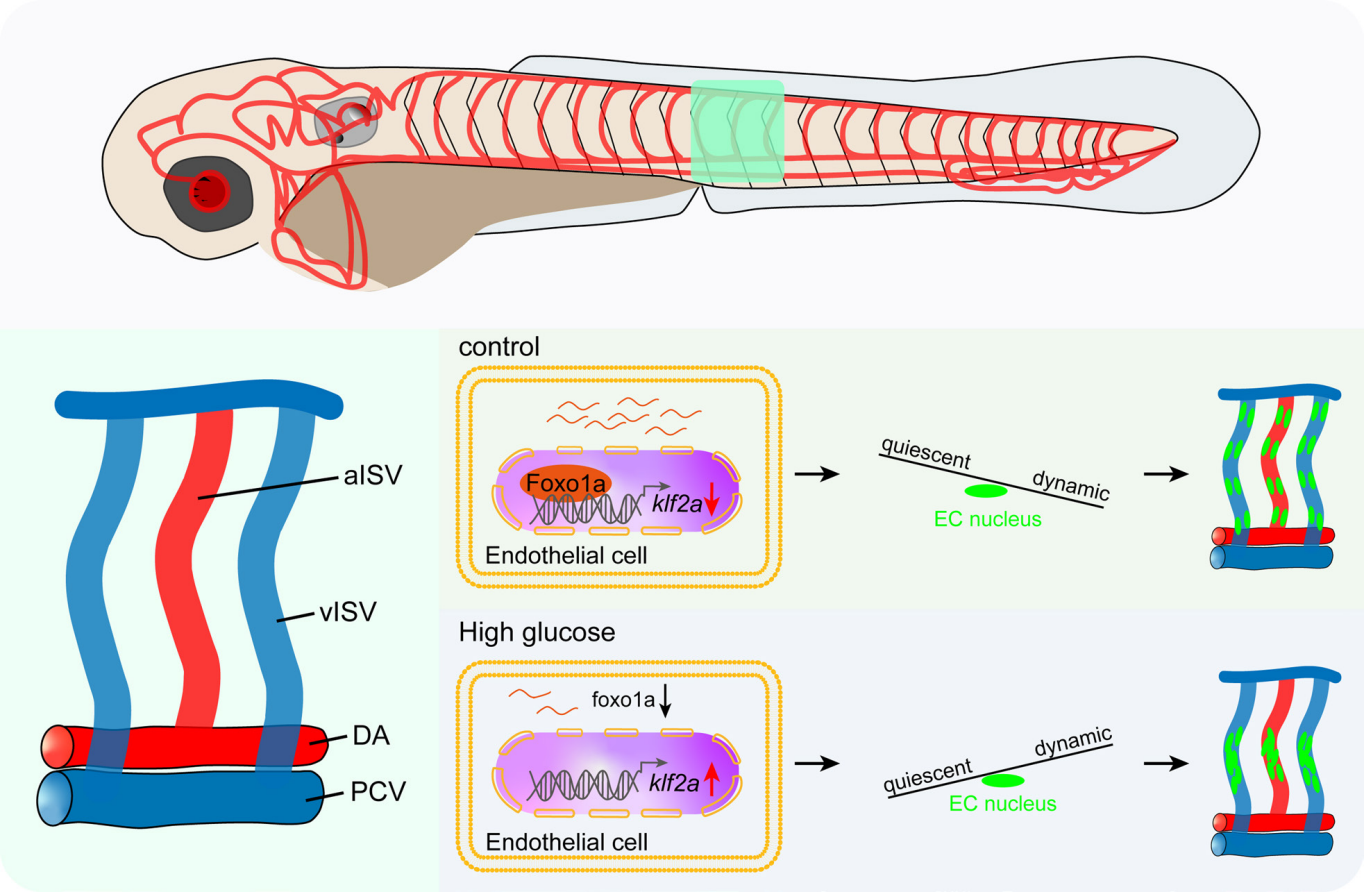
**Working model of high glucose treatment induced endothelial cell (EC) nuclei aggregation in intersegmental vessels of zebrafish embryos.** A indicates dorsal aorta; aISV, arterial intersegmental vessel; EC, endothelial cell; Foxo1a, forkhead box protein O1a; klf2a, Krüppel-like factor 2a; PCV, posterior cardinal vein; and vISV, venous intersegmental vessel.

Diabetes-induced microvascular complications are long-term damages that affect small blood vessels. The causes of this condition involve a combination of decreased eNOS (endothelial NO synthase), oxidative stress, and the production of advanced glycosylation end products.^49–51^ For instance, diabetic retinopathy, a common microvascular complication, initially manifests through the existence of microaneurysms.^52,53^ Microaneurysms are tiny areas of swelling in the blood vessels and consist of several crowded nuclei. In this study, we observed an aggregation of vascular endothelial nuclei in the ISVs of zebrafish embryos induced by high glucose, similar to the crowded nuclei in microaneurysms (Figure 7). Further examination revealed hyperactivity and excessive migration of ECs. Thus, we infer that the aggregation of endothelial nuclei may result from the aberrant migration of these cells. It is known that both proliferation and migration are essential for angiogenesis. However, excessive proliferation and migration can be detrimental and lead to various malignant conditions such as diabetic vascular complications, atherosclerosis, restenosis, and cancers.^54,55^ Our findings provide new evidence on how high glucose levels affect vascular status by regulating EC behavior.

Previous studies have shown that FOXO1 plays a significant role in regulating gluconeogenesis and glycogenolysis through the insulin signaling pathway.^56,57^ Activation of FOXO1 due to insulin resistance exacerbates diabetic cardiomyopathy, while FOXO1 deficiency can effectively reduce heart failure and restore cardiac function in db/db and high-fat diet mice.^58^ Sustained activation of FOXO1 leads to a thin and hyperbranched vascular network with fewer ECs.^59–61^ Endothelial-specific FOXO1 deficiency promotes vascular growth in the adipose tissue of obese mice.^62^ Accumulating evidence indicates that FOXO1 is also involved in the development and progression of diabetes. Inhibition of FOXO1 promotes angiogenesis and recovery of EC function in diabetic mice.^63^ Some studies have also demonstrated the role of FOXO1 in microvascular complications.^64–66^ In zebrafish, *foxo1* has 2 duplicate genes, *foxo1a* and *foxo1b*. Although the role of *foxo1a* and *foxo1b* in zebrafish vasculature is not well understood, a recent study has reported that *foxo1* is involved in embryonic vascular development. ^67^We found that *foxo1a* was significantly downregulated after high glucose treatment (Figure S7), which caused excessive angiogenesis in the ISVs of high glucose–treated embryos.^30^

In this study, our investigation used techniques such as single-cell sequencing analysis and ChIP-PCR to provide evidence of EC nuclei aggregation in the ISVs of high glucose–treated embryos. The single-cell sequencing data showed that *slc2a1a*, a glucose transporter, was dramatically changed after high glucose treatment. However, overexpressing *slc2a1a* in zebrafish had no effect on the vascular development (Figure S8). In our previous work, we found that deficiency of Foxo1a induced excessive angiogenesis. Using AS1842856 to inhibit Foxo1a indicated that Foxo1a is also involved in EC nuclei aggregation (Figure 4B). We also examined *marcksl1a*, which we found to be a downstream effector of Foxo1a in the process of excessive angiogenesis. The results suggested that *marcksl1a* does not regulate EC nuclei aggregation in ISVs (Figure S9). Finally, we found that this regulation is achieved through the upregulation of *klf2a* expression. Notably, KLF2 is a crucial transcriptional factor in ECs that controls NO production, which is important in renal disease.^68^ Dysregulation of KLF2 is responsible for cardiac microvascular disease in diabetes, specifically related to defects in monocyte adhesion and migration in ECs.^69^ Previous research has identified the reciprocal regulation of *KLF2* by FOXO1 in HUVECs, suggesting a potential mechanism for diabetic endothelial dysfunction.^42^ In our study, we utilized ChIP-PCR and dominant-negative experiments to confirm the involvement of *klf2a* and its interaction with Foxo1 in ECs in the progress of EC nuclei aggregation caused by high glucose treatment.

To date, existing literature has indicated a correlation between endothelial dysfunction in microvascular complications and increased oxidative stress, reduced NO release, activation of protein kinase C, and increased production of inflammatory factors.^70^ However, understanding the underlying cellular and molecular mechanisms of diabetic microvascular complications remains limited. This study is the first to demonstrate that high glucose treatment induces EC nuclei aggregation and abnormal migration of ECs in vivo. Moreover, it has been determined that this phenotype induced by high glucose is mediated through the *foxo1a-klf2a* axis. In conclusion, our findings provide a new perspective on the mechanism of microvascular complications in hyperglycemia.

## Article Information

### Sources of Funding

This study was supported by grants from the National Natural Science Foundation of China (81870359 and 92368104).

### Disclosures

None.

### Supplemental Material

Tables S1–S3

Figures S1–S9

Videos S1–S3

Major Resources Table
